# Working Memory and Activity Diversity in Older Adults: Examining Within- and Between-Person Associations

**DOI:** 10.1093/geroni/igaf122.1604

**Published:** 2025-12-31

**Authors:** Johanna Drewelies, Denis Gerstorf, Sandra Duezel, Ilja Demuth, Ulman Lindenberger, Rachel Koffer, Soomi Lee

**Affiliations:** Humboldt Universität zu Berlin, Berlin, Berlin, Germany; Humboldt University Berlin, Humboldt University Berlin, Berlin, Germany; Charité - Universitätsmedizin Berlin, Berlin, Berlin, Germany; Charité - Universitätsmedizin Berlin, Berlin, Berlin, Germany; Max Planck Institute for Human Development, Berlin, Berlin, Germany; Arizona State University, Phoenix, Arizona, United States; The Pennsylvania State University, University Park, Pennsylvania, United States

## Abstract

Cognitive function and activity diversity have been linked, yet it remains unclear whether this association holds at the daily level. This study examined associations between daily working memory and within- and between-person activity diversity in older adults. Data were collected six times per day for seven days from 127 participants in Berlin (Mage = 76.59, range: 67–88). Participants reported their activity engagement across social, physical, mental, productive, household chores, self-care, and leisure domains; a daily activity diversity score was calculated using Shannon’s entropy. Multi-level models adjusted for age, gender, education, morbidity, perceptual speed, and negative affect and loneliness. Results showed that, at the between-person level, individuals with greater overall activity diversity exhibited better working memory. However, at the within-person level, on days when individuals engaged in more varied activities than usual, working memory was lower. Negative affect and loneliness moderated these associations: higher negative affect buffered the within-person effect of activity diversity on working memory, while loneliness amplified it. These findings suggest that although engaging in diverse activities may support working memory over the long term, daily fluctuations in activity diversity could be cognitively taxing. The interplay between emotional states and activity diversity highlights the complexity of daily cognitive functioning in older adults.

